# Porta hepatis lymphnode mimicking biliary atresia: A case report

**DOI:** 10.1016/j.ijscr.2024.110040

**Published:** 2024-07-19

**Authors:** Elisamia Ngowi, Juliana Kwayu, Abduel Kitua, Mohamedraza Ebrahim, Naomi Mwamanenge, Yaser Abdallah

**Affiliations:** a Department of Paediatrics and Child Health, Aga Khan Hospital Tanzania, P.O. Box 2289, Dar Es Salaam, Tanzania; b Department of Paediatrics and Child Health, Aga Khan University, P.O. Box 38129, Dar Es Salaam, Tanzania; c Department of Surgery, Aga Khan University, P.O. Box 38129, Dar Es Salaam, Tanzania

**Keywords:** Cholestatic jaundice, Unconjugated hyperbilirubinemia, Biliary atresia, Case report

## Abstract

**Introduction:**

Cholestasis is the impairment of normal bile flow causing accumulation of bile salts, lipids, and bilirubin in blood which presents as Jaundice. Jaundice beyond 2 weeks of age is rare in infancy with worldwide incidence of 1 in 2500 live births. Biliary atresia is the most common extra hepatic cause of cholestasis in late neonatal and infancy period. Cholestasis and hyperbilirubinemia cause irreversible brain and liver damage if not diagnosed and treated early.

**Case presentation:**

A 3-week-old neonate presenting with progressive yellowish discoloration of eyes and skin. Explorative laparotomy found anatomically normal liver and biliary tree, but a lymph node obstructing the common bile duct.

**Discussion:**

This case was particularly unique as history of illness and initial investigations were suggestive of biliary atresia. However, the patient had lymph nodes with no history of any triggers to lymphadenopathy. It is a rare case of obstruction of biliary flow in this age group.

**Conclusion:**

Despite biliary atresia being the commonest cause of obstructive jaundice in infancy, it is important to rule out other causes like lymph nodes obstructing the biliary tree.

## Highlights

• Cholestatic jaundice is rare in infancy and biliary atresia should be ruled out promptly as the most common cause.
• It is important to do biopsy before exploratory laparotomy to minimize unnecessary surgical procedures.
• Intrahepatic lymph node can mimic biliary atresia.

## 1 Introduction

Jaundice in the newborn refers to the yellow discoloration of the sclera, mucous membranes, skin, tissues and bodily fluids, as a result of excess circulating serum bilirubin [1]. It can be either direct or indirect. Most newborns have indirect hyperbilirubinemia due to several causes which resolves within the first two weeks of life. Jaundice that is persistent beyond two weeks in full term neonates and 3 weeks in premature neonates is not normal and it warrants further investigation to identify the cause. The most common cause of jaundice beyond 2 weeks of life is cholestasis [2].

Cholestasis refers to obstruction of the normal excretion of bile from the liver resulting in abnormal accumulation of the bile salts, bilirubin, and lipids in blood [3]. The worldwide incidence of cholestasis is 1 in 2500 livebirths [4]. A study in East Africa reported a mortality rate of approximately 10 % in infants presenting with prolonged jaundice [5]. In infancy, cholestatic jaundice is mostly attributed to biliary atresia in approximately 25 to 40 % of cases, followed by genetic disorders and prematurity [6].

Early diagnosis and intervention with Kasai procedure in Biliary atresia reduces morbidity in infancy and childhood, hence the importance to rule out Biliary atresia as early as possible [7]. However, causes of cholestasis in neonates and infants can be grouped into extrahepatic and intrahepatic with Biliary atresia as the most common extra hepatic cause. The common intrahepatic causes of cholestasis may include α1-antitrypsin deficiency, viral infections and genetic cholestatic disorders, like Alagille's syndrome and different types of progressive familial intrahepatic cholestasis. There have been reported cases of choledocholithiasis as a cause of cholestatic jaundice in neonates [8]. Despite these documented causes, many will have unknown causes [2]. Here we describe an unusual cause of cholestasis, initially thought to be due to biliary atresia but intra operative findings were of porta hepatis lymph nodes obstructing biliary flow along the biliary tract. Remarkably, this represents a very rare cause of neonatal cholestasis and has only been described predominantly in adult patients with metastasis or invasion to the hepatic hilum and granulomatous inflammatory conditions with adenopathy [9].

This case report has been reported in line with the Surgical Case Report Guidelines (SCARE) [10].

## 2 Case presentation

A 3-week-old male neonate was admitted due to yellowish discoloration of the eyes and skin beginning at 2 weeks of age. Discoloration progressed gradually from the eyes to the peripheries. The patient passed pale-dry stool and dark, yellowish-coloured urine. There were no symptoms of fever, abdominal distension, vomiting, or petechiae rash. There were no signs of encephalopathy or seizures. The infant was delivered by emergency caesarean section to a primigravida mother at 37 weeks of gestation due to non-reassuring foetal status and was treated for presumed sepsis after birth. He had a low birth weight of 1.97 kg. The baby's mother is blood group O positive and had pregnancy-induced hypertension but no history of intrapartum infection.

On admission, Total Serum bilirubin was 143 umol/L (normal range: <80 umol/L), Direct Serum Bilirubin was 123 umol/L (normal range: <20 umol/L) indicating cholestasis. Haemoglobin levels were 18.1 g/dL (normal range:13.5–21 g/dL) with normal differentials, and total White blood cell count of 10.6 × 10^9^/L (normal range: 10–20 × 10^9^/L) with normal differentials. The infant has Blood Group O-Positive. There were elevated liver function parameters like Serum SGOT/AST of 356 IU/L (normal range: 0–40 IU/L), Serum SGPT/ALT of 93 (normal range: 0–40 IU/L), Serum Alkaline Phosphatase of 1170 IU/L (normal range: <250 IU/L), Serum Gamma GT of 77 IU/L (normal range: 8–61 IU/L) and low Total Protein of 43.9 g/L (normal range 53–89 g/L). Other investigations included INR of 1.25, urinalysis which was normal, screening for TORCHES infections were all negative, normal Thyroid Hormones levels, no pathogen growth detected in blood and urine culture, negative HIV ELISA test and no DNA detected on PCR test done.

**Fig. 1:**
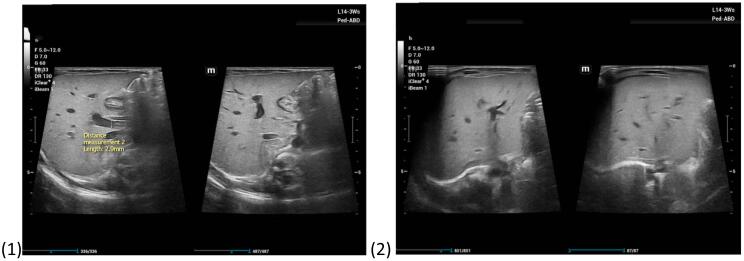
Ultrasound images on admission.

Provisional diagnosis of Biliary atresia was made, and initial abdominal ultrasound showed Persistently contracted gall bladder with ratio of gall bladder length (14 mm) to width (2.1 mm) measuring 7. Biliary radicals were not dilated but anterior triangular cord was measuring 9 mm (normal <4 mm) in liver, features are highly suggestive of biliary atresia as seen on Fig. 1. Cranial Ultrasound, Chest X-ray and ECHO were normal, done to rule out any associated anomalies. Commensurate with these findings, a Hepatobiliary Iminodiacetic acid (HIDA) scan was done and showed features suggestive of Biliary Atresia as shown in Fig. 3.

Explorative laparotomy was done with a Kocher incision. After thorough inspection, the anatomy of the liver, gallbladder and biliary tree were all normal in structure Fig. 6. There was a lymph node compressing the common bile duct at the porta hepatis. Excision biopsy of the lymph node and biopsy of the liver were taken for histopathological analysis.

The Liver fragment histology revealed Sections showing lymphocytes with congested sinuses, but no giant cells or bile plugs identified Fig. 4. There were neutrophil infiltrates and scattered lymphocytes hence a diagnosis of liver subacute hepatitis. The lymph node sections showed a lymphoid tissue partly lined by a fibrous capsule, dilation of sinuses with histiocytes and congested blood vessels hence a diagnosis of porta hepatis reactive lymphadenitis was made Fig. 5.

**Fig. 2:**
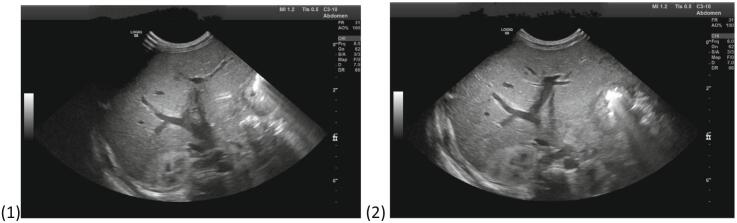
Abdominal ultrasound images 1-week post-surgery.

The infant underwent post-surgery care as per hospital protocol with an uneventful post operative stay. Abdominal ultrasound done 1 week post explorative laparotomy showed no evidence of biliary tree dilatation Fig. 2.

**Fig. 3:**
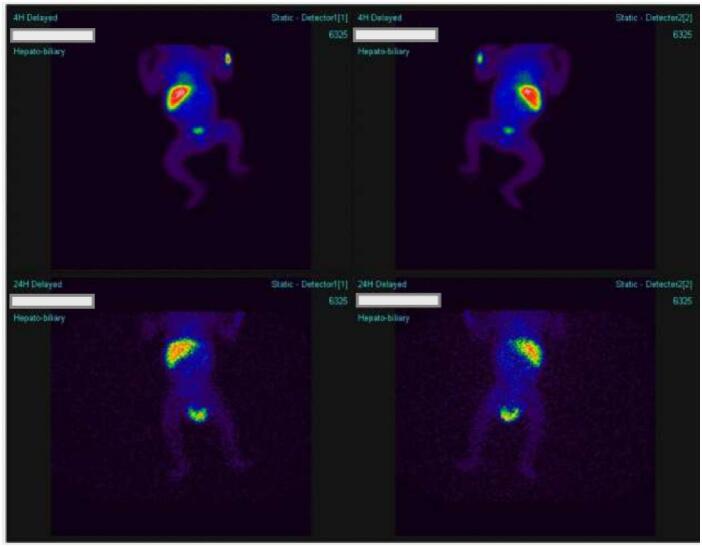
A dynamic HIDA SCAN study was acquired for 60 min, and static images were obtained at 1 h and 24 h. There was good hepatocellular function with features in keeping with Biliary Atresia.

**Fig. 4:**
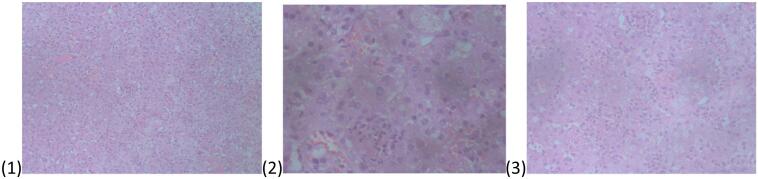
Slides of liver tissue.

**Fig. 5:**
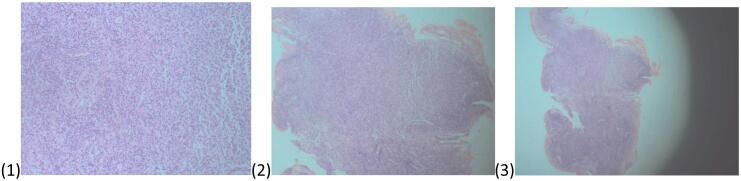
Histopathological images from lymph nodes.

**Fig. 6:**
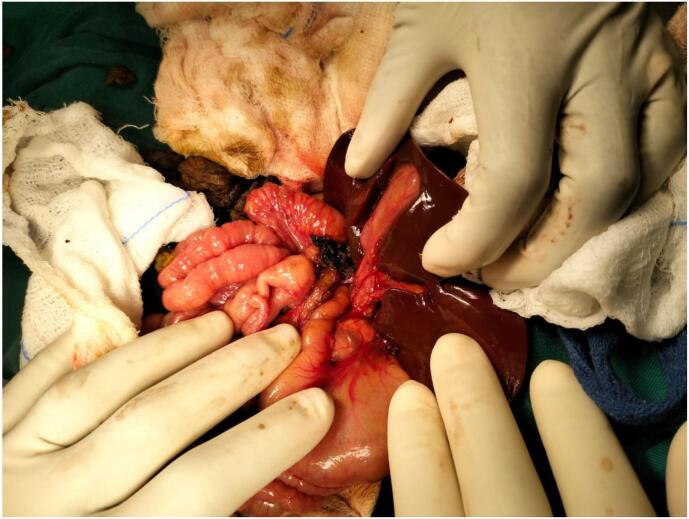
Intra operative image showing a biliary tree, part of the liver and surrounding structures.

The infant was discharged with reduced Serum Bilirubin levels, whereby Total Serum Bilirubin was 88 umol/L and Direct Bilirubin was 69 umol/L.

The infant was under regular clinic follow up and last Total Serum Bilirubin was a 11 umol/L and Direct Serum Bilirubin of 7 umol/L. Other liver function tests were within normal ranges at the last clinic visit.

## 3 Discussion

Cholestasis in the newborn period requires extensive investigations and timely management. There are several causes for cholestasis in this age group which can be either Hepatobiliary or metabolic causes. Hepatobiliary causes can be physical or mechanical barriers to flow of bile in the biliary tract. Obstructive lesions such as gallstones, choledochal cysts, annular pancreas and rarely hematologic malignancies have been reported to be some of the Hepatobiliary causes. Hepatocellular causes of conjugated hyperbilirubinemia include idiopathic neonatal hepatitis, viral hepatitis, Wilson's disease, alpha-1-anti trypsin, Dubin-Johnson syndrome, inborn errors of metabolism and rotor syndrome [11]. However, our patient did not meet the criteria for most of the above common aetiologies of cholestasis from the initial evaluation and investigations were pointing towards biliary atresia.

Any neonate with jaundice after 2 weeks of life is recommended to be evaluated appropriately with measurement of total and direct serum bilirubin. If serum direct bilirubin is elevated, further evaluation is warranted to rule out the common causes of conjugated hyperbilirubinemia. Surgical exploration and intraoperative Cholangiography remain the gold standard for diagnosing biliary atresia and have been applied for many years. Imaging and liver histopathology are important to evaluate bile duct patency to exclude treatable surgical conditions, since Kasai procedure is less likely to benefit infants if performed after 3 months of age [6,12]. Initial non-invasive evaluation limits the rates of unnecessary laparotomies ensuring only cases with high suspicion for biliary atresia are subjected to surgical exploration and cholangiography [13]. Obstructive jaundice has been reported in an adult with tuberculosis to be caused by liver hilar lymph node that was resulting into narrowing of common bile duct [14]. However, lymph node obstruction to bile flow in neonates or infancy is a very rare occurrence and there are no well described cases. Our patient was found to have lymph node obstructing biliary flow in contrast to the initial diagnosis of biliary atresia. The anatomy of the biliary tree was normal as seen intraoperatively. This underscores the challenge with non-invasive diagnostic evaluation in neonatal cholestasis and the low sensitivity and positive predictive value of HIDA scan to differentiate mechanical biliary obstruction of other cause other than Biliary atresia [13,15].

Furthermore, in assessing cholestasis in an infant, the examiner should consider extrahepatic signs like dysmorphism, poor growth, neurocutaneous manifestations, and pulmonary symptoms. The presence of hepatomegaly may indicate biliary atresia, splenomegaly may indicate haemolytic or glycogen storage disorders, whereas atopic spleen or polysplenia may indicate biliary atresia splenic malformation (BASM). Presence of heart murmurs are indicative of a possible cardiac condition associated with hepatic failure. Stool and urine colour must be observed directly as acholic stools and dark urine indicate conjugated hyperbilirubinemia [3]. This holistic approach aids in selection of appropriate evaluation and diagnostic tests and limits evaluation time, allowing for early surgical intervention to address surgical extrahepatic causes of neonatal cholestasis [16]. Our patient did not exhibit any physical signs of dysmorphism and continued to gain weight as expected. He had a normal cardiac evaluation. However, he presented with pale stool as evaluated using the stool colour chart, as well as dark urine.

## 4 Conclusion

This case report highlights porta hepatis lymphadenopathy as a very rare cause of extrahepatic biliary flow obstruction in infants. When evaluating images of the biliary tree in infants with cholestasis, clinicians should consider the possibility of lymph node obstruction. Moreover, early surgical exploration and intra-operative cholangiography should be considered for cases with clinical and diagnostic evaluation findings suggesting extrahepatic biliary obstruction.

## Consent for publication

Written informed consent was obtained from the patient's parents/legal guardian for publication and any accompanying images. A copy of the written consent is available for review by the Editor-in-Chief of this journal on request.

## Ethical approval

Not required for case reports at our hospital for single case reports.

## Funding

No funds were needed to publish this case.

## Author contribution

Elisamia Ngowi was involved in the conception, study design, acquisition, and interpretation of data, and drafting of the manuscript.

Juliana Kwayu, Mohamedraza Ebrahim and Naomi Mwamanenge reviewed literature of the research work.

Abduel Kitua was involved in interpretation of clinical data, literature review and manuscript writing.

Yaser Abdallah supervised and reviewed the whole research work.

All authors read and approved the final manuscript.

## Guarantor

Elisamia Ngowi, is the main guarantor of this research work. Elingowi80@gmail.com.

## Research registration number

This case report is not a First in Man study.

## Declaration of generative AI and AI-assisted technologies in the writing process

The corresponding author only applied generative AI called Word Tune to correct grammatical errors in only 2 paragraphs of this manuscript and nothing else. The author reviewed, edited the content, and takes full responsibility for publication.

## Conflict of interest statement

The authors declare that they have no competing interests.

## Data Availability

The datasets of the present study are available from the corresponding author upon request.
